# Note upon the Existence of Rhythmical Contractions in the Excretory Ducts of the Principal Glands in Birds

**Published:** 1859-08

**Authors:** E. Brown-Sèquard


ART. VII.—*Note upon the Existence of Rhythmical Contractions in the Excretory Ducts of the principal Glands in Birds.* By DR. E. BROWN-SÉQUARD. Translated by LUCIEN DAMAINVILLE, Md. Student.

In 1849, M. Cl. Bernard (*Comptes Rendus de la Societé de Biol.* vol. i. p. 171)—announced that he had ascertained that the biliary and pancreatic canals contract in a rhythmical manner, in pigeons and fowls. While studying these rhythmical movements, I have had occasion to find several facts that I have communicated to the Biological Society, in June, 1856, but which have not been published till now. (These facts, however, are mentioned in the Paris correspondence of the "Medical Times and Gazette,' August, 1856, p. 121.)

1. The spermatic canals in adult birds, principally in spring and summer, have rhythmical movements as regular as those of the heart, averaging from 10 to 20 a minute.

2. The rhythm of the movements of the left vas deferens, differs from the one on the right.

3. The contraction commences at the origin of the duct, and propagates itself gradually to its termination, in the same way as in the pancreatic and biliary ducts, and in the ureters.

4. The rhythm is different in these four species of excretory ducts.

5. The rapidity of the rhythmical movements of these different ducts, the same as that of the heart, is increased for a while when the animal is asphyxiated.

6. The destruction of the cerebro-spinal centre is not followed by an immediate cessation of the rhythmical movements of these four species of ducts; from which it follows that they do not depend directly upon the action of this nervous centre.

In the mammalia, except the ureters, where everybody knows that rhythmical movements exist, I have never seen such movements in the various excretory ducts; and it is probable that Müller, who speaks of the rhythmical movements of the biliary ducts, as an acknowledged fact (*Manuel de Physiologie Ed. Littré,* 1851, p. 273, vol. i.), has seen these movements—if he has seen them—only in birds.

I will add, that in the large birds I have seen rhythmical respiratory movements in the trachea and large bronchial tubes.

